# Association Between Gut Dysbiosis and Sepsis-Induced Myocardial Dysfunction in Patients With Sepsis or Septic Shock

**DOI:** 10.3389/fcimb.2022.857035

**Published:** 2022-03-14

**Authors:** Yu Chen, Fu Zhang, Xin Ye, Jing-Juan Hu, Xiao Yang, Lin Yao, Bing-Cheng Zhao, Fan Deng, Ke-Xuan Liu

**Affiliations:** ^1^ Department of Anesthesiology, Nanfang Hospital, Southern Medical University, Guangzhou, China; ^2^ Department of Anesthesiology, Jinshan Branch of Fujian Provincial Hospital, Fuzhou, China; ^3^ Department of Anesthesiology, Fujian Provincial Clinical Medical College, Fujian Medical University, Fuzhou, China; ^4^ Department of Clinical Medicine, Fujian Medical University, Fuzhou, China

**Keywords:** intensive care unit, sepsis, gut microbiota, sepsis-induced myocardial dysfunction, *Klebsiella variicola*

## Abstract

**Objective:**

Sepsis-induced myocardial dysfunction (SIMD) seriously affects the evolution and prognosis of the sepsis patient. The gut microbiota has been confirmed to play an important role in sepsis or cardiovascular diseases, but the changes and roles of the gut microbiota in SIMD have not been reported yet. This study aims to assess the compositions of the gut microbiota in sepsis or septic patients with or without myocardial injury and to find the relationship between the gut microbiota and SIMD.

**Methods:**

The prospective, observational, and 1:1 matched case–control study was conducted to observe gut microbiota profiles from patients with SIMD (n = 18) and matched non-SIMD (NSIMD) patients (n = 18) by 16S rRNA gene sequencing. Then the relationship between the relative abundance of microbial taxa and clinical indicators and clinical outcomes related to SIMD was analyzed. The receiver operating characteristic (ROC) curves were used to evaluate the predictive efficiencies of the varied gut microbiota to SIMD.

**Results:**

SIMD was associated with poor outcomes in sepsis patients. The beta-diversity of the gut microbiota was significantly different between the SIMD patients and NSIMD subjects. The gut microbiota profiles in different levels significantly differed between the two groups. Additionally, the abundance of some microbes (*Klebsiella variicola*, Enterobacteriaceae, and *Bacteroides vulgatus*) was correlated with clinical indicators and clinical outcomes. Notably, ROC analysis indicated that *K. variicola* may be a potential biomarker of SIMD.

**Conclusion:**

Our study indicates that SIMD patients may have a particular gut microbiota signature and that the gut microbiota might be a potential diagnostic marker for evaluating the risk of developing SIMD.

## Introduction

Sepsis is an infection that triggers an abnormal host response and leads to systemic inflammatory response syndrome, which has very high morbidity and fatality rates (Yao et al., 2020; Miao et al., 2021). Importantly, the current rapid development of medical technology has not effectively reduced the mortality of patients with sepsis, which has become a major issue that plagues human health. Recent studies have found that once sepsis is combined with myocardial damage, it can aggravate the evolution of the disease, increase the risk of multiple organ failure and death, and seriously affect the prognosis of the patient. It was shown that about 40% to 50% of sepsis patients have cardiac insufficiency, and sepsis-induced myocardial dysfunction (SIMD) is an increasingly recognized form of transient cardiac dysfunction characterized by decreased ventricular systolic and diastolic function, and/or reduced response to volume resuscitation (Zhang et al., 2020; Liu and Chong, 2021). A historical cohort study of 388 patients hospitalized with severe sepsis or septic shock demonstrated that even isolated right ventricular dysfunction is associated with worse long-term survival (Vallabhajosyula et al., 2017). Furthermore, another study showed that elevated high-sensitivity cardiac troponin I (hs-cTnI) levels not only increase the death rates during the first 14 days but also significantly reduce the 1-year survival rate of patients (Frencken et al., 2018). Therefore, effective prevention of SIMD, early prediction, and diagnosis and treatment of SIMD may be reliable strategies to reduce the injury and even mortality of patients with sepsis.

There are a large number and a wide variety of symbiotic microbiota living in the intestines of humans, collectively termed the gut microbiota (Gill et al., 2006; Deng et al., 2021a; Deng et al., 2021b). The gut microbiota has formed a close relationship with its host over the course of long-term evolution and has been considered the most important microecosystem living in symbiosis with the body (Schmidt et al., 2018; Shanahan et al., 2021; Siwczak et al., 2021; Deng et al., 2022). Existing evidence suggests that a diverse and balanced intestinal microbiota can enhance the host’s immunity to intestinal and systemic pathogens, and disturbing this balance is likely to increase the susceptibility to sepsis. On the other hand, studies have shown that sepsis and its treatment severely affect the composition of the intestinal microbiota, but the clinical outcome caused by these effects needs further research. Studies have reported that when compared to a healthy population, pathogenic microorganisms in septic patients can overwhelm the indigenous species of the gut microbiota, thus resulting in the loss of beneficial microbial species (Wolff et al., 2018). Besides, previous studies had shown that the abundance of pathogenic species, such as *Enterococcus* spp., was differentially increased in sepsis patients who died, indicating that the gut ecosystem is expected to be a prognostic marker for patients with septic complications (Shimizu et al., 2011; Agudelo-Ochoa and Valdés-Duque, 2020). It has been confirmed that the dysbiotic gut microbiota may be identified as a key player in the process of sepsis. Interestingly, recent studies also indicated that both the types of the microorganisms and their relative abundance may participate in the onset and progression of cardiovascular diseases such as coronary artery disease, hypertension, or heart failure (Franzosa et al., 2015; Gózd-Barszczewska et al., 2017; Kamo and Akazawa, 2017; Zuo and Li, 2019). However, whether the gut microbiota is involved in the SIMD has not yet been reported. In this preliminary study, we aimed to describe the gut microbiota profiles in SIMD patients in the intensive care unit (ICU) and try to shed light on the role of gut microbiota composition as a contributing factor in the evolution of these patients and its influence on the severity of the myocardial injury, the function of the heart, and ICU stay time. Additionally, we wished to explore the potential predictive biomarker of the gut microbiota for SIMD.

## Material and Methods

### Participant and Controls

A prospective observational, single-center, 1:1 matched case–control preliminary study was conducted in Fujian Provincial Hospital. The study was approved by the Fujian Provincial Hospital ethics committee (Institutional Review Board, K2021-02-005; registration number, ChiCTR2100050499). Informed consent was obtained from each enrolled patient accompanied by at least a family member or proxy. We prospectively enrolled all consecutive adult (≥18 years) patients presenting to the ICU with sepsis or septic shock between September 2021 and December 2021. The following exclusion criteria were applied: 1) inflammation involving the heart, including myocarditis, pericarditis, and endocarditis; 2) active diagnoses directly relating to myocardial dysfunction, such as acute myocardial infarction, unstable arrhythmia, and post-cardiopulmonary resuscitation status; 3) patients with significant underlying cardiac conditions, such as congenital heart disease and valvular heart disease; 4) patients who underwent cardiac surgery within 2 months; 5) pregnant and lactating patients; 6) unable to complete the echocardiograms in a timely manner; and 7) unwillingness to participate in the study or unexpected discharge.

SIMD cases and non-SIMD (NSIMD) controls were frequency matched (1:1) on four variables using incidence density sampling. Specifically, one non-myocardial dysfunction control was randomly selected for each SIMD case from the source population according to the four matched variables, including age within 5 years, sex, infection site, and presence of cardiovascular comorbidity.

### Recruitment

**Figure 1:**
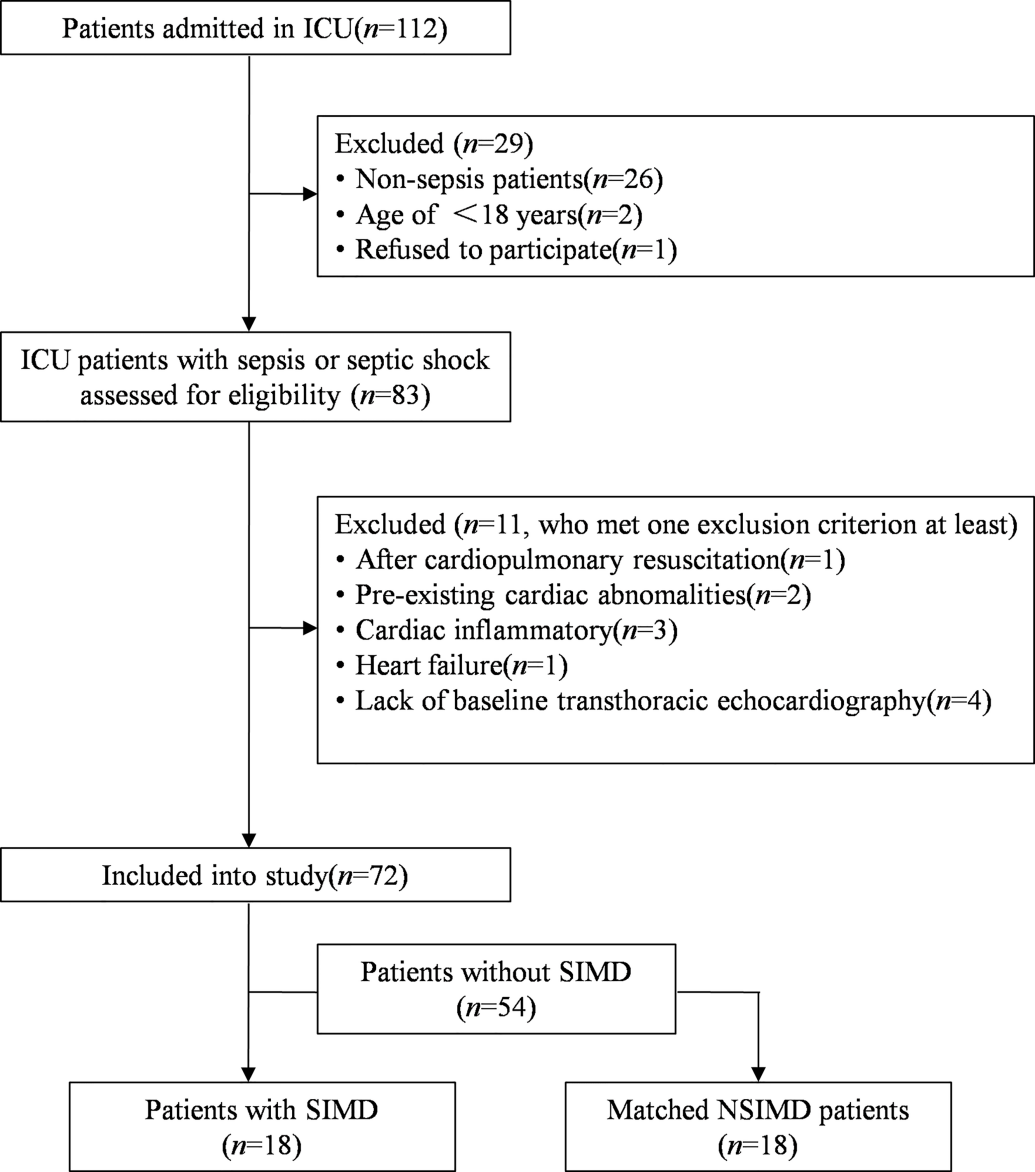
Flowchart of this matched case–control study. SIMD, sepsis-induced myocardial dysfunction; NSIMD, non-SIMD.

A total of 112 consecutive patients were admitted to the ICU from September 2021 to December 2021, of whom 83 adult patients were diagnosed with sepsis or septic shock. However, 13 patients were excluded because they met at least one exclusion criterion, and finally 72 patients were included in our study. Among the patients included in the study, a total of 18 patients met the diagnostic criteria for SIMD, and the incidence of SIMD was 25.0%. Another 18 patients who did not experience SIMD but were enrolled in this study served as controls by matched variables. A patient recruitment flowchart is shown in **Figure 1**.

### Variable Definitions

Sepsis and septic shock were defined according to the Third International Consensus Definitions for Sepsis and Septic Shock (Sepsis-3) (Singer et al., 2016). Sepsis was defined as life-threatening organ dysfunction caused by a dysregulated host response to infection. Clinical diagnosis was represented by an increase in the Sequential [Sepsis-related] Organ Failure Assessment (SOFA) score of 2 points or more. Septic shock was defined as a subset of sepsis in which underlying circulatory and cellular metabolism abnormalities were profound enough to substantially increase mortality, referred to as a state of persisting hypotension requiring vasopressors to maintain mean arterial pressure (MAP) ≥ 65 mmHg and having a serum lactate level >2 mmol/L (18 mg/dl) despite adequate volume resuscitation. The severity of illness was assessed on the day of admission to the intensive care unit. SIMD was diagnosed established by both hs-cTnI levels and bedside transthoracic echocardiography (TTE). Specifically, hs-cTnI levels were monitored, and TTE was performed in the first 24 h of admission to ICU and if the parameter is higher than 0.04 ng/ml (Kim et al., 2020) (a cutoff value that represents the 99th percentile reported in the healthy population). Meanwhile, an echocardiogram confirmed that the left ventricular ejection fraction (LVEF) <50% and/or ≥10% in the patient’s initial EF assessed on admission (Jeong et al., 2018). If and only if these two conditions are satisfied, the SIMD was definitely diagnosed.

### Fecal Sample Collection and Gut Microbiota Profiling

Fecal samples were acquired from the patients by swabs of the rectum at the time of ICU admission. Samples with a cotton applicator were then put into test tubes with RNAlater (Invitrogen, Vilnius, Lithuania), an RNA stabilization solution, and the volume ratio of RNAlater to the sample was 1:5–10 (Flores et al., 2015). All samples were stored at −80°C until further processing.

Microbiome DNA was extracted using the HiPure Soil DNA Kits (Magen, Guangzhou, China) according to the manufacturer’s protocols. The 16S rRNA genes of the hypervariable V3–V4 regions were amplified by PCR with the primers 341-F, 5′-CCTACGGGNGGCWGCAG-3′ and 806-R, 5′-GGACTACHVGGGTATCTAAT-3′ (Liu et al., 2021). The amplicons were evaluated with 2% agarose gels and purified using the AxyPrep DNA Gel Extraction Kit (Axygen Biosciences, Union City, CA, USA) according to the manufacturer’s instructions. Sequencing libraries were generated using SMRTbell™ Template Prep Kit (PacBio, Menlo Park, CA, USA) following the manufacturer’s recommendation. The library quality was assessed with Qubit 3.0 Fluorometer (Thermo Fisher Scientific, Waltham, MA, USA) and FEMTO Pulse system (Agilent Technologies, Santa Clara, CA, USA). The purified amplicons were pooled in equimolar quantities and subjected to paired-end sequencing (2 × 250) on an Illumina HiSeq 2500 Platform (Illumina Inc., San Diego, CA, USA) according to standard protocols.

### Statistical Analysis

The normal distributed continuous variables were presented as means ± SD or percentages, while non-normal distributed continuous variables were reported as median with interquartile ranges (Q1–Q3). The categorical variables were compared using the chi-square test or Fisher’s exact test. The continuous variables were compared using the Mann–Whitney rank test or a Student’s t-test, depending on the distribution of the data. Statistical analyses were performed using IBM SPSS Statistics version 23.0 (IBMCorp., Armonk, NY, USA.). A *p*-value < 0.05 was considered to be statistically significant.

The gut microbiota sequence was mainly analyzed by the QIIME (version 1.9.1) and R project Vegan package (version 2.5.3) (Caporaso et al., 2010). For the analyses among groups, Venn diagram-based analyses were performed in the R project to compare the taxon relative abundance at all levels between the two groups (Chen and Boutros, 2011). The alpha-diversity index such as the Chao1 richness estimator, Shannon diversity index, and Simpson diversity index were calculated to investigate fecal microbiota community richness, and alpha index comparison among these two groups was performed with the Kruskal–Wallis test and Tukey’s honestly significant difference (HSD) test. The beta-diversity analysis was applied to evaluate the structural variation of microbial communities based on principal coordinate analysis (PCoA) of unweighted UniFrac distances and visualized *via* non-metric multidimensional scaling. Adonis (also called permutational multivariate ANOVA (PERMANOVA)) and analysis of similarity (ANOSIM) test were also performed to indicate the total microbial composition difference of the two groups. The Kyoto Encyclopedia of Genes and Genomes (KEGG) pathway analysis of the operational taxonomic units (OTUs) was inferred using Tax4Fun (version 1.0) or PICRUSt (version 2.1.4) (Langille et al., 2013). The association between gut microbiota composition and the clinical indicators related to myocardial injury were conducted by Spearman’s rank correlation test. The area under the receiver operating characteristic (ROC) curve (AUC) was used to designate the ROC effect.

## Results

### Patient Characteristics

The characteristics of the patients are listed in **Table 1**. There were no significant differences between SIMD cases and NSIMD controls for any of the four matched variables (age, sex, infection site, and presence of cardiovascular comorbidity), suggesting a successful matching procedure. Besides, the blood culture showed no differences between the SIMD and NSIMD groups. However, the pathogens were identified to be quite low in each group (7/18 in both the SIMD and NSIMD groups), which may due to the high rate of antibiotics used before blood cultures (Lim et al., 2021). The SOFA score (10.5 [7.5–13.3] *vs.* 4.0 [4.0–7.0], *p* < 0.001) and incidence of septic shock (61.1% *vs.* 16.7%, *p* = 0.015) were significantly higher in the SIMD group than NSIMD subjects. Results of all laboratory parameters other than hs-cTnI were comparable, and no statistically significant differences could be established between the two groups. Consistent with the study definitions, a significantly higher serum hs-cTnI (0.35 [0.13–0.64] *vs*. 0.02 [0.01–0.07], *p* < 0.001) was found in SIMD patients compared with the NSIMD subjects. With regard to the echocardiographic parameters, there was a significant decline versus baseline in LVEF in the SIMD group, while no differences were observed in the NSIMD group. The comparison of clinical outcomes between patients with SIMD and those without SIMD is presented in **Table 2**. The subjects with SIMD exhibited significantly higher requirements of vasoactive drugs (72.2% *vs*. 16.7%, *p* = 0.002) and longer ICU length of stay (14.5 [10.5–21.0] *vs*. 9.5 [7.75–11.0], *p* = 0.001). The 28-day mortality and requirements of mechanical ventilator were comparable between the two groups.

**Table 1:** Matched patient characteristics.

Characteristics	SIMD (n = 18)	NSIMD (n = 18)	*p*-Value
**Age**	65.0 [48.8–74.8]	65.0 [49.3–77.0]	0.882
**Male**	10 (55.6)	10 (55.6)	1.000
**Medical history**			
Hypertension	8 (44.4)	10 (55.6)	0.740
Diabetes mellitus	3 (16.7)	4 (17.0)	>0.999
CKD	3 (16.7)	3 (17.0)	1.000
COPD	1 (5.6)	0 (0.0)	>0.999
**Sources of infection**			
Pulmonary	6 (33.3)	7 (38.9)	>0.999
Urinary	4 (22.2)	3 (16.7)	>0.999
GI	4 (22.2)	4 (22.2)	>0.999
HBP	2 (11.1)	2 (11.1)	1.000
Other	2 (11.1)	2 (11.1)	1.000
**Bacteremia**			
Gram positive	3 (16.7)	3 (16.7)	1.000
Gram negative	4 (22.2)	3 (16.7)	>0.999
Fungus	0 (11.1)	1 (11.1)	>0.999
**SOFA score**	10.5 [7.5–13.3]	4.0 [4.0–7.0]	<0.001
**Septic shock**	11 (61.1)	3 (16.7)	0.015
**Laboratory**			
WCC (×10^3^/μl)	10.45 [6.98–20.38]	11.00 [9.50–13.80]	0.983
Hemoglobin (g/dl)	108.17 ± 26.99	109.94 ± 21.13	0.831
Platelet (×10^3^/μl)	131.5 [65.25–150.0]	159.0 [100.5–268.0]	0.054
PT-INR	1.23 ± 0.22	1.20 ± 0.20	0.643
hs-cTnI (ng/ml)	0.35 [0.13–0.64]	0.02 [0.01–0.07]	<0.001
BNP (pg/ml)	8071.74 ± 10927.36	3643.04 ± 8408.33	0.190
BUN (mg/dl)	248.44 ± 26.916	195.25 ± 301.81	0.591
Creatinine (mg/dl)	12.55 [6.05–17.75]	8.7 [4.88–20.88]	0.615
**Transthoracic echocardiography**			
Base LVEF (%)	59.33 ± 4.89	59.78 ± 5.21	0.793
LVEF (%)	39.28 ± 5.02	58.22 ± 4.78	<0.001

Data presented as mean ± SD, median [IQR] or number (%).

SIMD, sepsis-induced myocardial dysfunction group; NSIMD, non-SIMD group; CKD, chronic kidney disease; COPD, chronic pulmonary disease; GI, gastrointestinal; HBP, hepato-biliary-pancreas; SOFA, Sequential Organ Failure Assessment; WCC, white cell count; hs-cTnI, high-sensitivity cardiac troponin I; PT-INR, prothrombin time—International Normalization Ratio; BNP, brain natriuretic peptide; BUN, blood urea nitrogen; LVEF, left ventricular ejection fraction; IQR, interquartile range.

**Table 2:** Comparison of clinical outcomes between the SIMD and NSIMD groups.

Clinical Outcomes	SIMD (n = 18)	NSIMD (n = 18)	*p*-Value
Requirement of ventilator	5 (27.8)	3 (16.7)	0.691
Requirement of vasoactive drugs	13 (72.2)	3 (16.7)	0.002
ICU length of stay	14.5 [10.5–21.0]	9.5 [7.75–11.0]	0.001
28-day mortality	5 (27.8)	2 (11.1)	0.402

Data presented as median [IQR] or number (%).

ICU, intensive care unit; IQR, interquartile range.

### Comparison of Bacterial Populations and Microbial Diversity

**Figure 2:**
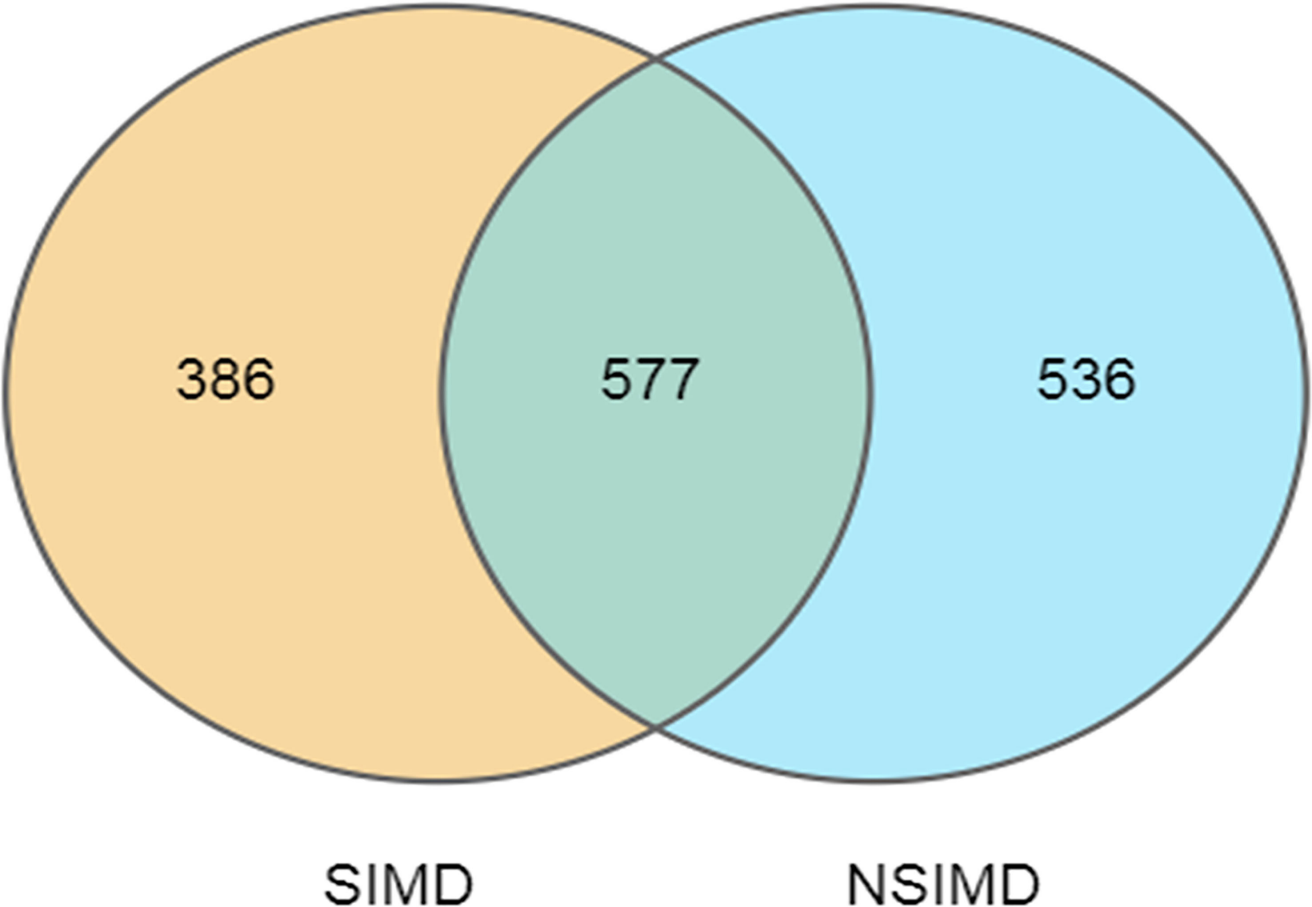
Venn diagram of the unique and shared OTUs between two groups. Fecal microbiota richness was significantly decreased in the SIMD group. OTUs, operational taxonomic units; SIMD, sepsis-induced myocardial dysfunction.

**Figure 3:**
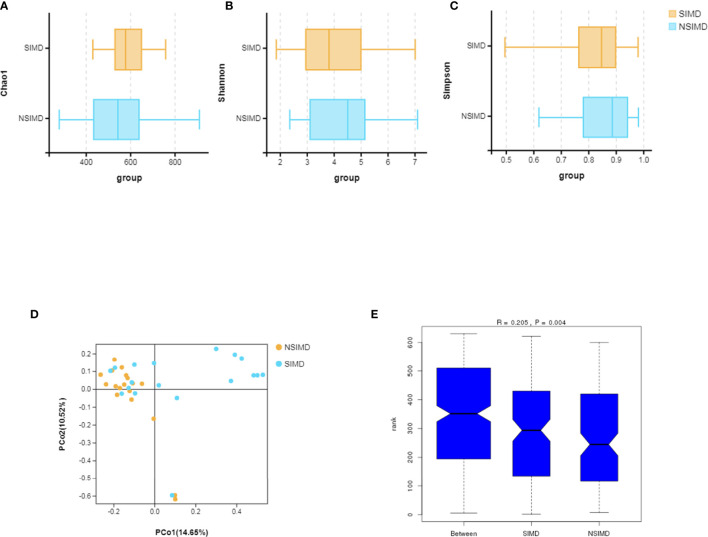
Comparisons of the microbial diversity between the SIMD and NSIMD groups. **(A–C)** No difference in alpha-diversity between the two groups estimated by Chao1 index, Shannon index, and Simpson index (*p* = 0.192, *p* = 0.355, *p* = 0.279, respectively). **(D)** Beta-diversity was calculated using weighted UniFrac by principal coordinate analysis (PCoA), indicating a symmetrical distribution of fecal microbial community among all the samples. **(E)** Global community was significantly different using ANOSIM test (R = 0.205, *p* = 0.004). SIMD, sepsis-induced myocardial dysfunction; NSIMD, non-SIMD; ANOSIM, analysis of similarity.

Fecal samples that were collected from all the recruited patients were sequenced on an Illumina HiSeq sequencer. A total of 3,458 OTUs (97% identity) were observed across all samples. Among this, the SIMD cohort had 386 unique OTUs, while the NSIMD group had 536 OTUs, with 577 OTUs shared between the two groups, and the Venn diagram suggests that patients without SIMD presented with a higher gut microbiota richness (**Figure 2**). The microbial diversity of fecal samples was the assessment by the intra-group comparison (alpha-diversity) and inter-group comparison (beta-diversity). Taking into account alpha-diversity parameters evaluated such as Chao1 (*p*_Wilcoxon_ = 0.192) (**Figure 3A**), Shannon index (*p*_Wilcoxon_ = 0.355) (**Figure 3B**), and Simpson index (*p*_Wilcoxon_ = 0.279) (**Figure 3C**), we found that there were no meaningful differential distributions of diversity descriptors between the two groups. Beta-diversity assessed by PCoA based on the OTU indicated that the microbial community structure differed significantly among the groups (PERMANOVA, *p* = 0.002) (**Figure 3D**). ANOSIM assessed by weighted UniFrac demonstrated that there were significant differences in the gut microbiota among groups (R = 0.205, *p* = 0.004) (**Figure 3E**).

### Characteristics and Alterations of Microbial Composition

**Figure 4:**
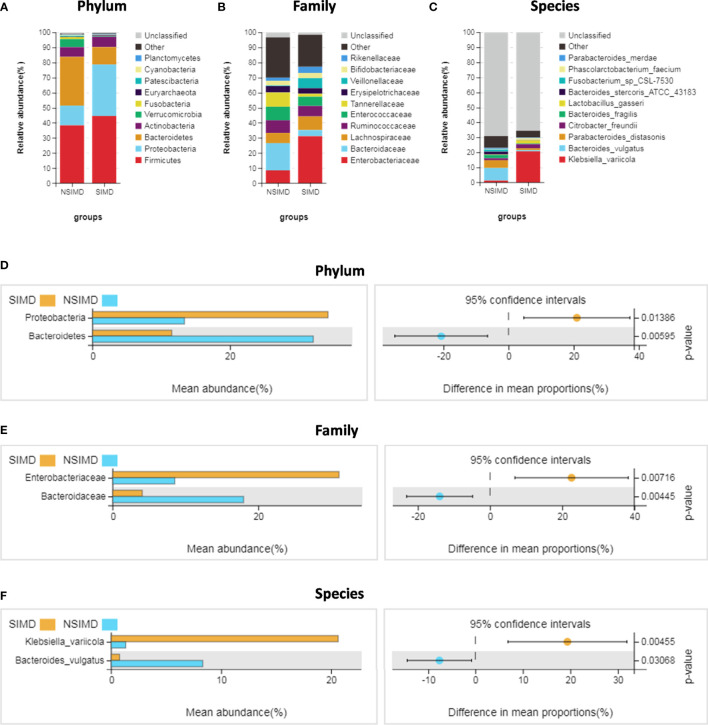
Distribution of intestinal microbiota and species analysis of differences between two groups. **(A**–**C)** A cylindrical accumulation map at the phylum **(A)**, family **(B)**, species **(C)** levels. The relative abundance of the 12 most abundant bacteria in subjects. **(D)** At the phylum level, the SIMD group had a higher relative abundance of Proteobacteria and a lower relative abundance of Bacteroidetes. **(E)** At the family level, the SIMD group had a higher relative abundance of Enterobacteriaceae and a lower relative abundance of Bacteroidaceae. **(F)** At the species level, the SIMD group had a higher relative abundance of *Klebsiella variicola* and a lower relative abundance of *Bacteroides vulgatus*. The graph on the right shows the difference in confidence levels between groups. SIMD, sepsis-induced myocardial dysfunction.

We compared the intestinal microbiota profiles in each group. The distribution of intestinal microbiota at different levels was significantly different (**Figures 4A–C**). At the phylum level, the SIMD group had a higher relative abundance of Proteobacteria (*p* = 0.014) and a lower relative abundance of Bacteroidetes (*p* = 0.006) than the NSIMD group; besides, the SIMD group also had a higher F/B ratio than the NSIMD group (**Figure 4D**). At the family level, a significantly higher proportion of Enterobacteriaceae (*p* = 0.007) and a lower proportion of Bacteroidaceae (*p* = 0.004) was found in SIMD patients as compared to the NSIMD subjects (**Figure 4E**). At the species level, the microbial abundance of *Klebsiella variicola* (*p* = 0.005) was significantly higher and that of *Bacteroides vulgatus* (*p* = 0.031) was significantly lower in the SIMD group as compared to the NSIMD group (**Figure 4F**).

### Functional Changes in Microbiomes Between Groups

**Figure 5:**
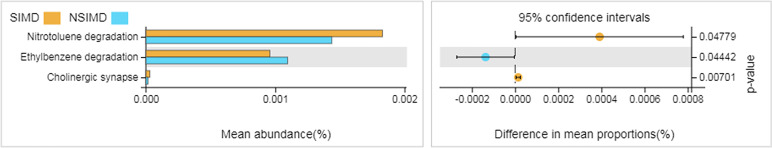
Functional pathway analysis of gut microbiome between two groups. Microbial functions were predicted using Tax4Fun at the third level of the KEGG pathway and statistically analyzed by Welch’s t-test between groups. The graph on the right shows the difference between the confidence levels of the groups. KEGG, Kyoto Encyclopedia of Genes and Genomes.

The different functional pathways were predicted between the SIMD and NSIMD groups. 16S rDNA sequencing data were analyzed using Tax4Fun at the third level, and a total of 284 KEGG modules were mapped. Compared with NSIMD fecal samples, the abundance of microbiota in SIMD patients was associated with pathways involved in nitrotoluene degradation, cholinergic synapse, and a reduction of ethylbenzene degradation (**Figure 5**).

### Relationships Between Clinical Indicators, Clinical Outcomes, and Gut Microbiota

**Figure 6:**
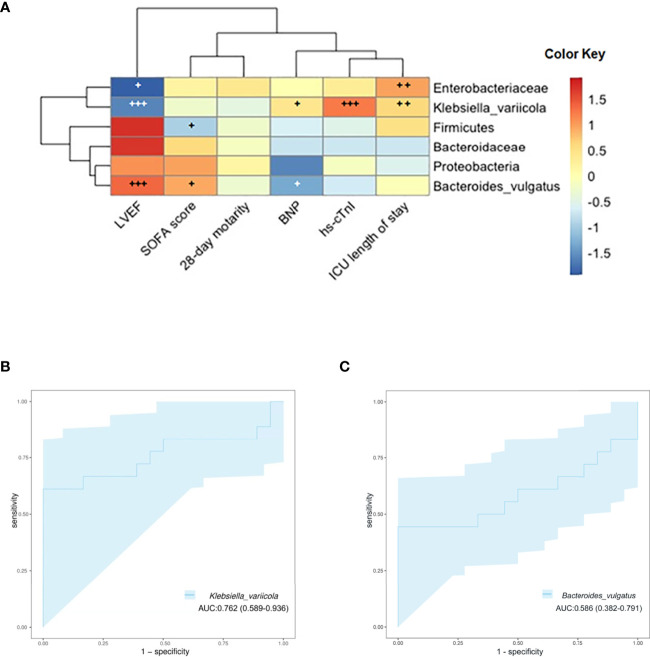
**(A)** Associations of specific microbiota in different levels with clinical characteristics. The heat map of Spearman’s rank correlation coefficients between the gut microbiota and clinical indicators (+, *p* < 0.10; ++, *p* < 0.05; +++, *p* < 0.01; LVEF, left ventricular ejection fraction; SOFA, Sequential Organ Failure Assessment; BNP, brain natriuretic peptide; hs-cTnI, high-sensitivity cardiac troponin I). **(B)** ROC curve showing the ability of *Klebsiella variicola* to predict SIMD. **(C)** ROC curve showing the ability of *Bacteroides vulgatus* to predict SIMD. ROC, receiver operating characteristic; SIMD, sepsis-induced myocardial dysfunction.

The associations of clinical indicators and clinical outcomes with the gut microbiota were also assessed. We found that the difference in the gut microbiota may pose a potential reason for these clinical phenomena through Spearman’s rank correlation analysis. The relative abundance of *K. variicola* was positively correlated with hs-cTnI and ICU length of stay but negatively with LVEF (*p* < 0.05). The amount of Enterobacteriaceae was positively correlated with ICU length of stay (*p* < 0.05). And the abundance of *B. vulgatus* was positively correlated with LVEF (*p* < 0.05) (**Figure 6A**). By this investigation, we found that in those with significantly altered microbiota, the abundance of *K. variicola* showed a strong correlation with worth clinical indicators and clinical outcomes induced by SIMD. Simultaneously, the severity of SIMD symptoms was inversely correlated with the abundance of *B. vulgatus*. To evaluate whether *K. variicola* or *B. vulgatus* could be used to distinguish the SIMD subjects from sepsis and septic shock patients, ROC curve was performed, and the abundance of *K. variicola* (AUC 0.762, 95% CI: 0.569–0.936) (**Figure 6B**) presented a significantly higher diagnostic accuracy as compared with the abundance of *B. vulgatus* (AUC 0.586, 95% CI: 0.382–0.791) (**Figure 6C**) in predicting SIMD.

## Discussion

In this study, we characterized the composition of the gut microbiome in ICU patients with SIMD and sepsis subjects without myocardial injury. Moreover, associations of the gut microbiota with clinical parameters and the outcomes of these sepsis patients were also investigated, and we aimed to find a novel and non-invasive biomarker for SIMD. In addition, we found that the taxonomic composition and inter-group microbial diversity of gut microbiota taxonomic composition and inter-group microbial diversity were significantly different between the two groups. Gut microbiota distribution at different levels was also significantly different. Moreover, a specific species named *K. variicola* was abundant in the SIMD group compared to NSIMD patients, and we also revealed that the relative abundance of *K. variicola* was correlated with clinical indicators and clinical outcomes in included subjects. Notably, this study indicated that any alteration regarding the proper balance of species inhabiting the human gut could have an impact on myocardial injury in sepsis or septic shock populations. Eventually, it may contribute to the development of more targeted interventions from a comprehensive perspective.

As shown in the results, the phenomenon of myocardial injury has been associated with poor outcomes in sepsis patients. It may lead to an increased dose of vasoactive medication, and a longer duration of ICU stay. Moreover, the 28-day mortality of SIMD patients was also elevated as compared to the NSIMD subjects; although this difference was not significant, it might be related to the small sample size. These discoveries were in line with previous studies, suggesting that the myocardial dysfunction will worsen the prognosis of sepsis or septic patients (Frencken et al., 2018; Kim et al., 2020). According to this finding, we should pay more attention to the frequent complications of sepsis. Early discovery, early diagnosis, and early proactive intervention may improve the prognosis of the sepsis or septic patients effectively.

In this present study, we found no apparent differences in terms of alpha-diversity descriptors of the gut microbiota between the SIMD group and the NSIMD group. However, the beta-diversity in line with the ANOSIM assessment, which indicates the inter-group diversity, has a significant difference. These results suggested that sepsis or septic patients with SIMD have an intestinal microbial community structure that is distinct from those without SIMD. Previous studies showed that sepsis conditions could drive changes in the microbial community structure, and this fragile community structure of gut microbial in turn provides optimal conditions for the development of diseases (Valle Gottlieb and Closs, 2018; Agudelo-Ochoa and Valdés-Duque, 2020).

In the current study, the Tax4Fun analysis revealed that three KEGG pathways significantly differed in the abundance of the gut microbiota between these two groups. Therein, nitrotoluene degradation and ethylbenzene degradation pathways are more likely to be enriched as compared to cholinergic synapses. Previous studies indicated that aerobic bacteria or aerotolerant anaerobe such as *Achromobacter* sp. and *Pseudarthrobacter chlorophenolicus* probably take part in the aerobic degradation of nitroaromatic compounds (Hudcova et al., 2011; Lamba et al., 2021; Okozide and Adebusoye, 2021). In addition, Haak and colleagues observed that a loss of the anaerobic intestinal environment and an overgrowth of aerobic pathobionts are capable of causing invasive diseases (Haak and Argelaguet, 2021). Hence, nitrotoluene degradation pathway was found to be enriched in the gut microbiota of SIMD patients. More recently, research suggests that benzene, toluene, ethylbenzene, and xylene (BTEX) seem to be important factors contributing to cardiovascular disease and were associated with the risk of mortality from heart disease (Ran et al., 2018; Li et al., 2022). Thus, the gut microbiota in the NSIMD group, which helps to enhance the ethylbenzene degradation, may contribute to the attenuation of myocardial damage.

Our results also demonstrated that the microbial composition in SIMD patients was clearly different from that of the NSIMD subjects. Specifically, the phylum Proteobacteria increased and the phylum Bacteroidetes decreased in the SIMD group. In accordance with a previous study (Wan et al., 2018), a significantly higher proportion of Proteobacteria was associated with a poor prognosis in patients with sepsis or septic. Besides, the SIMD group showed a higher F/B ratio that usually indicates gut microbiota dysbiosis, which can make patients susceptible to hospital-acquired infections or organ failure (McDonald et al., 2016; Hu et al., 2019). Beyond this, a few microbes in different levels such as Enterobacteriaceae, Bacteroidaceae, *K. variicola*, and *B. vulgatus* were also found to differ significantly between the groups. Previous studies have shown that the family Enterobacteriaceae can instigate inflammation to induce colitis (Garrett et al., 2010) and may also contribute to the development of immune disorders in infants (Willers et al., 2020); taking these results into consideration, the significantly higher abundance of Enterobacteriaceae in SIMD group can suggest a status of gut flora dysbiosis that may lead to an exacerbated inflammatory response (Liu et al., 2015). Moreover, the species *B. vulgatus* was significantly enriched in the NSIMD group and was also negatively correlated with LVEF. This result suggests that *B. vulgatus* might have myocardial protective effects in sepsis or septic shock patients. The result was consistent with the previous study (Yoshida et al., 2018), which shows that *B. vulgatus* can be beneficial in attenuating coronary artery disease symptomology by inhibiting atherosclerosis.

Of note, we observed that *K. variicola* was the species with the most significant variation in abundance between the two groups, the relative abundance of *K. variicola* in the feces of SIMD patients was significantly higher than that of the NSIMD subjects. Furthermore, our Spearman’s correlation analysis showed that the abundance of *K. variicola* correlated well with several clinical indicators and clinical outcomes related to SIMD. To better understand whether the relative abundance of *K. variicola* is a reliable predictive biomarker to SIMD, the ROC curve was constructed. For the first time, herein, we found that *K. variicola* might be a novel potential biomarker by achieving a high accuracy (AUC 0.762). Previous studies have pointed out that *K. variicola* was first identified as a new bacterial strain in 2004 (Rosenblueth et al., 2004). As one of the subspecies within the *Klebsiella pneumoniae* complex, *K. variicola* has received much attention recently for their hypermucoviscosity and hypervirulence (Rodríguez-Medina et al., 2019; Morales-León and Opazo-Capurro, 2021; Nakamura-Silva et al., 2021). Recently research also found that the *K. variicola* strains have potential capacity to colonize in different tissues and cause infection (de Campos et al., 2021). Moreover, a previous study confirmed that the β-lactam antibiotic-resistant *K. variicola* strains can promote inflammation by inducing Th1 cells and inhibiting Treg (Lin et al., 2020). Therefore, we suggested that the role of *K. variicola* in host immunity may also be related to the risk of SIMD. These findings provide important evidence that gut microbiota-targeted biomarkers were potentially helpful in predicting the occurrence and prognosis of myocardial injury in patients with sepsis or septic shock and the treatment decisions.

However, several limitations of this study should be mentioned. First, the small clinical sample size may limit the interpretation of the results. Second, fecal sampling was collected only once for each patient and is therefore unable to capture the following changes. Third, functional validation experiments were not performed, and the effect of the altered abundance of *K. variicola* and other microbiota on the development of SIMD would need to be investigated further.

## Conclusions

To the best of our knowledge, this is the first study to demonstrate the flora microbiota characteristics in SIMD patients based on 16S rRNA gene sequencing technology. Our findings showed significant differences in microbial diversity, functional pathways, and microbial composition of the gut microbiota between the groups. Notably, we also found the alteration of *K. variicola* relative abundance might be helpful for predicting the occurrence and disease prognosis of SIMD. Our study provides a novel insight into the pathophysiological changes and pathogenesis of SIMD and may have important implications for identifying sepsis or septic patients at risk for the development of SIMD.

## Data Availability Statement

The data presented in the study are deposited in the NCBI repository, accession number PRJNA797231.

## Ethics Statement

The studies involving human participants were reviewed and approved by The Institutional Ethics Committee of Fujian provincial Hospital. The patients/participants provided their written informed consent to participate in this study.

## Author Contributions

YC and FD conceived and designed the project. XYe and LY participated in patient recruitment and data collection. XY and B-CZ analyzed the data and performed the statistical analysis. YC and FZ drafted the manuscript. K-XL and J-JH managed the manuscript preparation and coordination. All authors read and approved the final manuscript.

## Funding

This work was supported by grants from the Key Program of National Natural Science Foundation (81730058 to K-XL); National Natural Science Foundation of China (82172141 to K-XL); China Postdoctoral Science Foundation(2021M701611 to FD); President Foundation of Nanfang Hospital (2021C048 to FD); and Startup Fund for Scientific Research, Fujian Medical university (2020QH1155 to YC).

## Conflict of Interest

The authors declare that the research was conducted in the absence of any commercial or financial relationships that could be construed as a potential conflict of interest.

## Publisher’s Note

All claims expressed in this article are solely those of the authors and do not necessarily represent those of their affiliated organizations, or those of the publisher, the editors and the reviewers. Any product that may be evaluated in this article, or claim that may be made by its manufacturer, is not guaranteed or endorsed by the publisher.
